# Supplementary material for: Untargeted saliva metabolomics by liquid chromatography—Mass spectrometry reveals markers of COVID-19 severity
Source: PLoS One. 2022 Sep 22;17(9):e0274967. doi: 10.1371/journal.pone.0274967 (PMC9498978; doi:10.1371/journal.pone.0274967)
Supplement: S1 Fig — Principal component analysis of each patient sample (circles) and batch QC’s (squares), coloured according to extraction batch, showing no significant clustering of patient samples according to extraction batch. (DOCX) [file pone.0274967.s001.docx]

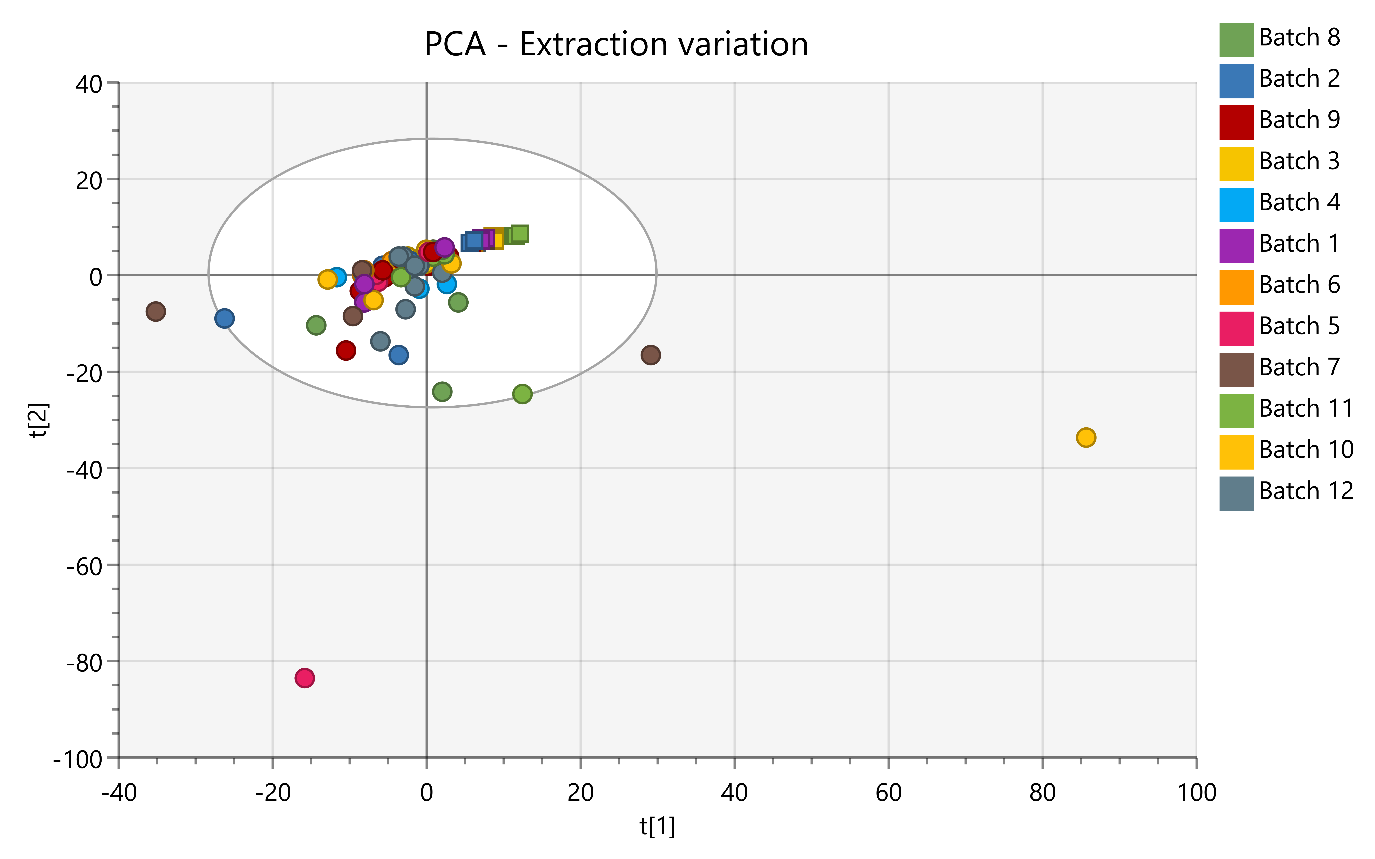


**S1 Fig:** Principal Component Analysis of each patient sample (circles) and batch QC’s (squares), coloured according to extraction batch, showing no significant clustering of patient samples according to extraction batch (square: QC ; circle: patients)
