# Supplementary material for: Untargeted saliva metabolomics by liquid chromatography—Mass spectrometry reveals markers of COVID-19 severity
Source: PLoS One. 2022 Sep 22;17(9):e0274967. doi: 10.1371/journal.pone.0274967 (PMC9498978; doi:10.1371/journal.pone.0274967)
Supplement: S2 Fig — (DOCX) [file pone.0274967.s002.docx]

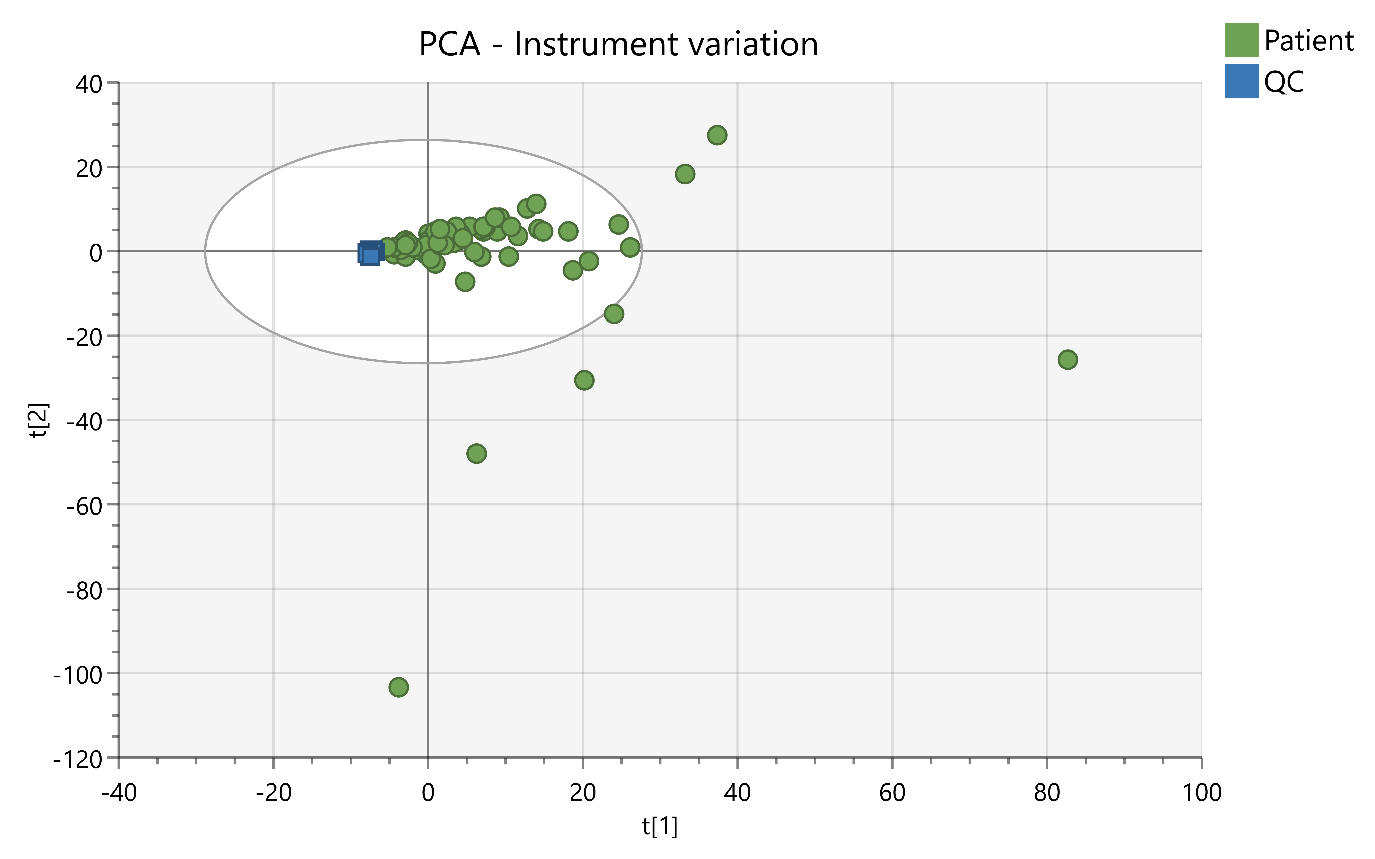


**S2 Fig:** Principal Component Analysis of each patient sample and run QC, showing low levels of QC variation according to position in the run sequence
