# Supplementary material for: Untargeted saliva metabolomics by liquid chromatography—Mass spectrometry reveals markers of COVID-19 severity
Source: PLoS One. 2022 Sep 22;17(9):e0274967. doi: 10.1371/journal.pone.0274967 (PMC9498978; doi:10.1371/journal.pone.0274967)
Supplement: S4 Fig — (DOCX) [file pone.0274967.s004.docx]

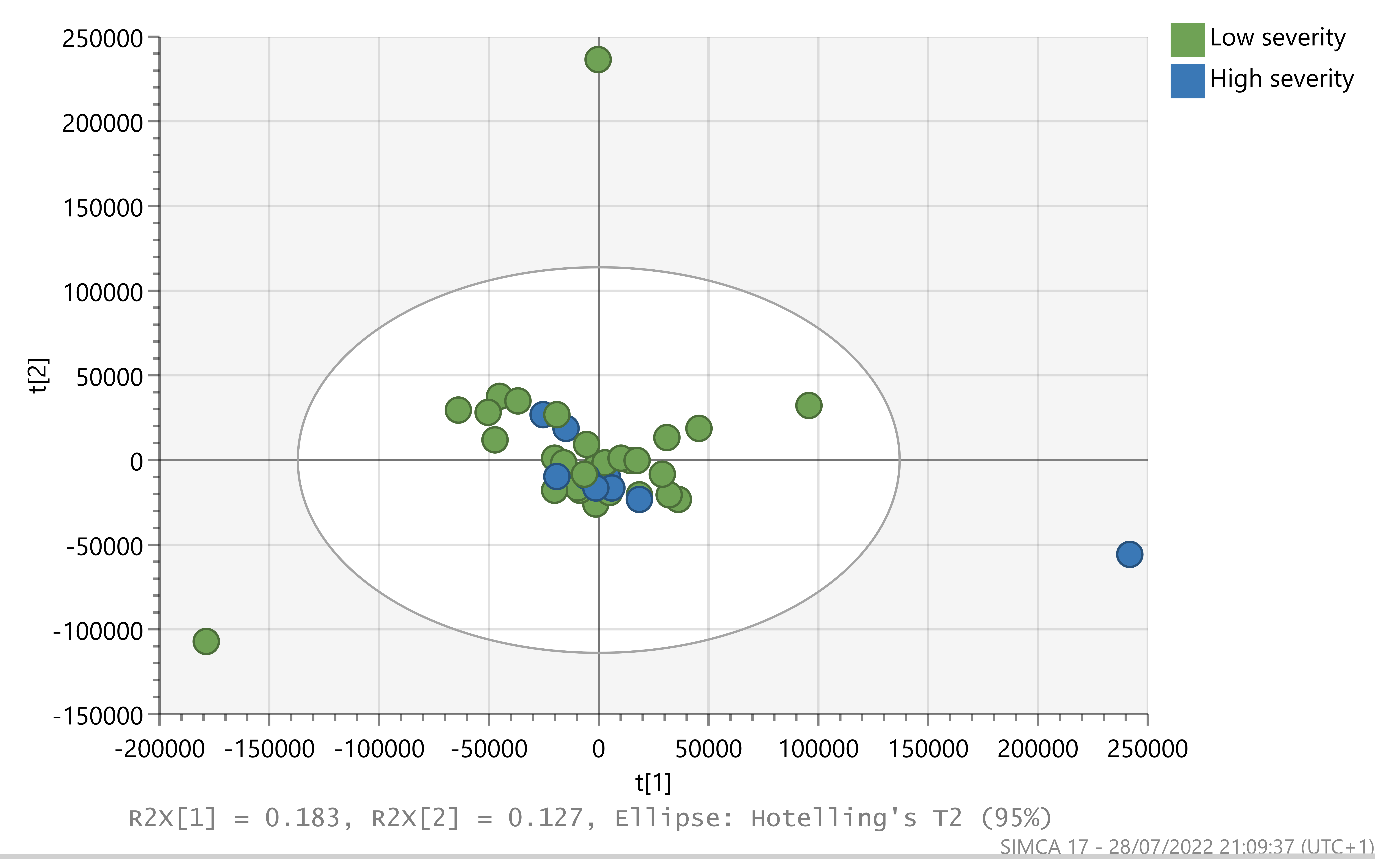


**S4 Fig:** Principal Component Analysis for 44 participants and 324 features, high severity / low severity, LC-MS analysis in positive mode.
