# Supplementary material for: Untargeted saliva metabolomics by liquid chromatography—Mass spectrometry reveals markers of COVID-19 severity
Source: PLoS One. 2022 Sep 22;17(9):e0274967. doi: 10.1371/journal.pone.0274967 (PMC9498978; doi:10.1371/journal.pone.0274967)
Supplement: S1 Table — (DOCX) [file pone.0274967.s005.docx]

**S1 Table:** Operating conditions of the mass spectrometer used in this research.

| Parameter | Operating condition |
| --- | --- |
| Spray voltage | 3.5 kV |
| Capillary temperature | 275 °C |
| S-lens RF level | 50 |
| Sheath gas flow rate | 40 |
| Aux gas flow rate | 0 |
| Scan range | 100 *m/z* to 1 000 *m/z* |
| Resolution | 70 000 |
| Polarity | Positive |
| AGC target | 10^6^ |
| Maximum inject time | 200 |
| MS/MS Parameter | **Operating condition** |
| Mode | Full Scan MS / dd-MS^2^ with inclusion lists |
| Resolution | 35 000 at m/z 200 |
| Loop Count | 6 |
| Intensity threshold | 5 * 10^4^ |
| Collision energy | 30 and 35 |
| Dynamic Exclusion | 5 seconds |
