# Supplementary material for: Untargeted saliva metabolomics by liquid chromatography—Mass spectrometry reveals markers of COVID-19 severity
Source: PLoS One. 2022 Sep 22;17(9):e0274967. doi: 10.1371/journal.pone.0274967 (PMC9498978; doi:10.1371/journal.pone.0274967)
Supplement: S2 Table — (DOCX) [file pone.0274967.s006.docx]

**S2 Table:** Features distinctive between COVID-19 positive and negative

| Feature | Fold Count | p-value |
| --- | --- | --- |
| C25 H36 N6 O6 | 0.52 | 0.0121 |
| DL-Phenylalanine | 1.39 | 0.0164 |
| C7 H10 N6 O2 | 1.35 | 0.0179 |
| 2-linoleoyl-sn-glycero-3-phosphoethanolamine | 1.85 | 0.0180 |
| C39 H71 N2 O16 P3 | 0.81 | 0.0226 |
| DC2810000 | 1.60 | 0.0231 |
| 179.8965 | 2.09 | 0.0251 |
| 1-Hexadecanoylpyrrolidine | 1.84 | 0.0287 |
| Taurine | 1.44 | 0.0292 |
| 2707 | 1.19 | 0.0363 |
| 148.0048 | 0.70 | 0.0379 |
| C47 H84 N9 O13 P3 | 0.73 | 0.0415 |
| C22 H36 N8 O9 | 1.39 | 0.0439 |
| 3721 | 1.70 | 0.0443 |
| butyl acrylate | 1.62 | 0.0470 |
| C2 H3 N3 O2 P2 | 1.25 | 0.0501 |
