# Supplementary material for: Untargeted saliva metabolomics by liquid chromatography—Mass spectrometry reveals markers of COVID-19 severity
Source: PLoS One. 2022 Sep 22;17(9):e0274967. doi: 10.1371/journal.pone.0274967 (PMC9498978; doi:10.1371/journal.pone.0274967)
Supplement: S3 Table — (DOCX) [file pone.0274967.s007.docx]

**S3 Table:** Features distinctive between COVID-19 high severity and low severity

| Feature | Fold Count | p-value |
| --- | --- | --- |
| C44 H74 N8 O16 | 0.50 | 0.0000 |
| C39 H67 N7 O15 | 0.55 | 0.0003 |
| C29 H56 N7 O11 P | 0.57 | 0.0013 |
| C42 H72 N8 O17 | 0.54 | 0.0038 |
| C49 H81 N9 O17 | 0.61 | 0.0148 |
| C37 H67 N6 O12 P3 | 0.51 | 0.0160 |
| C47 H84 N9 O13 P3 | 0.66 | 0.0178 |
| Valine | 0.48 | 0.0198 |
| C6 H13 N2 O6 P | 1.92 | 0.0237 |
| C9 H21 N O6 | 2.17 | 0.0241 |
| C17 H22 N4 O8 | 1.98 | 0.0247 |
| C8 H12 O5 P2 | 2.34 | 0.0254 |
| C2 H8 O5 P2 | 1.41 | 0.0258 |
| C60 H86 N10 O9 P2 | 0.65 | 0.0270 |
| C32 H48 N8 O9 | 0.47 | 0.0289 |
| Lys-phe | 0.48 | 0.0367 |
| N-{3-[(4-Acetamidobutyl)amino]propyl}acetamide | 0.59 | 0.0382 |
| Leucine | 0.67 | 0.0405 |
| C42 H71 N7 O15 | 0.44 | 0.0453 |
| 273.86553 | 1.29 | 0.0475 |
